# Adverse fetal and neonatal outcomes in pregnancies with confirmed Zika Virus infection in Rio de Janeiro, Brazil: A cohort study

**DOI:** 10.1371/journal.pntd.0008893

**Published:** 2021-01-04

**Authors:** Juliana P. Souza, Maria Dalva B. B. Méio, Laura Medeiros de Andrade, Mirza R. Figueiredo, Saint Clair Gomes Junior, Jose Paulo Pereira Junior, Elizabeth Brickley, Maria Elisabeth Lopes Moreira

**Affiliations:** 1 Instituto Nacional de Saúde da Mulher da Criança e do Adolescente Fernandes Figueira–IFF/Fundação Oswaldo Cruz–Fiocruz, Rio de Janeiro, Rio de Janeiro, Brazil; 2 Assistant Professor of Infectious Disease Epidemiology, Faculty of Epidemiology and Population, London School Hygiene and Tropical Medicine, London, United Kingdom; Fundacao Oswaldo Cruz, BRAZIL

## Abstract

**Objective:**

To analyze adverse fetal and neonatal outcomes of Zika virus infection by the timing of infection during pregnancy. Method: Cohort study of 190 pregnancies with 193 offspring with a positive RT-PCR test for Zika virus (March/2016 to April/2017).

**Results:**

Death or defects related to congenital Zika virus infection were identified in 37.3% of fetuses and newborns, and microcephaly in 21.4% of the newborns. The proportion of small for gestational age newborns was 21.9%. Maternal symptoms in the first trimester were significantly associated with the birth of newborns with microcephaly/cerebral atrophy, small for gestational age and with the deaths (one abortion, one stillbirth and the two neonatal deaths). Maternal infection during the second trimester was further associated with asymptomatic newborns at birth. The study showed that 58.5% of the offspring with microcephaly and / or cortical atrophy were small for gestational age, with an evident decrease in symptomatic offspring without microcephaly, 24.1%, and with only 9.1% in the asymptomatic group.

**Conclusion:**

This study showed that the earlier the symptoms appear during gestation, the more severe the endpoints. We found a higher percentage of small for gestational age newborns exposed to Zika virus early in gestation. We also found a group of apparently asymptomatic newborns with proven Zika infection, which highlights the importance of follow up studies in this population.

## Author summary

Zika virus infected pregnant women brought many challenges for the public health system because at the beginning of the outbreak there was no knowledge of the consequences of this disease. Microcephaly became the more common and threatening finding related to intrauterine Zika virus infection. Moreover, it was observed that there were others serious consequences of this disease during pregnancy, which were a burden for mothers and children. This study, following infected pregnant women, referred to our institution, enabled the medical professional team to improve their expertise in dealing with this new disease. Ours results showed a high frequency of intrauterine growth restriction, with babies being born small. Besides, it was found that the more severe cases occurred in women who were infected during the first trimester of gestation. Infections during the last half of the gestation more frequently leaded to babies born without symptoms. However, these results highlight the importance of following the babies born apparently asssymptomatic.

## Introduction

Congenital Zika virus (ZIKV) infections have the potential to severely affect fetal development and can lead to a wide range of adverse pregnancy and neonatal outcomes, including spontaneous abortion, fetal loss, low birth weight, and congenital malformations, especially in the central nervous system [1]. Despite some other studies involving pregnant women with confirmed ZIKV infection [2–6] gaps in knowledge are still found, especially regarding the broad spectrum of perinatal involvement of congenital ZIKV infection, ranging from apparently asymptomatic newborns to fetal and neonatal death.

Although the different methods used in the evaluation and classification of the newborn may have contributed to the differing risk estimates, it remains uncertain whether other biological or environmental co-factors could contribute to the observed heterogeneity [7]. Several studies to date have provided evidence that higher risks of adverse outcomes are associated with early, as compared to later, ZIKV infections during pregnancy [2,4,5].

Based on these findings, we hypothesized that earlier maternal infections may cause more deleterious embryonic or fetal compromise and, therefore, more severe repercussions for prenatal development. Thus, this study aimed to evaluate the adverse fetal and neonatal endpoints due to ZIKV congenital infection by timing of viral infection during gestation and to describe the range of abnormalities observed among the children with Congenital Zika Syndrome.

## Materials and methods

### Ethics statement

The study followed regulatory standards and guidelines established under Resolution N° 466/2012 of the National Health Council and demands from the IFF Research Ethics Committee. The subscribed number at CAAE 526756616000005269 referred to the Project “Vertical Exposure to Zika Virus and Its Consequences for Child Neurodevelopment” and registered in Clinical Trials.gov Identifier: NCT03255369. Written consent forms were obtained from the pregnant women before inclusion in the study.

### Study design

A cross sectional study was performed in a cohort of pregnant women exposed to ZIKV infection analyzing the perinatal repercussions in pregnancy considering the trimester of exposition (NCT03255369).

#### Study site

The study was conducted at the Obstetrics and Neonatology Services of the Fernandes Figueira National Institute of Women, Children and Adolescent Health/Oswaldo Cruz Foundation (IFF/FIOCRUZ), a public tertiary hospital located in Rio de Janeiro, Brazil. This hospital is a reference center for congenital malformations and has provided specialized care for the ZIKV-infected women and their children in the city of Rio de Janeiro since the onset of 2015–2017 epidemic.

### Population

The study population was recruited from a convenience sample of 194 pregnant women who were referred by public and private healthcare services after laboratory diagnosis, clinical symptoms, or evidence of fetal alteration during gestational ultrasound suggestive of ZIKV infection.

Pregnant women with acute febrile syndrome, cutaneous rash or fetal ultrasound with malformations suspected for Zika syndrome who tested positive by RT-PCR (urine or blood) were included in the study, as well newborn infants with some suspected malformations related do ZIKV who tested positive by RT-PCR.

### Exclusion criteria

The exclusion criteria were specific situations found in the study’s population unrelated to ZIKV congenital infection. One case of type II Arnold Chiari was excluded, as there was no certainty if the ZIKV infection occurred before or after the diagnostic of the malformation. Newborns with chromosomic alterations detected during fetal life by amniocentesis or at birth, were excluded from the original cohort. Also, all fetuses and newborns with laboratory-confirmed congenital infection by other TORCH [8] group agents were excluded at the admission in the original cohort.

### Exposure

The primary exposure was the trimester of the proven maternal ZIKV infection. Gestational trimesters were defined as: first trimester: up to 13 weeks and 6 days; second trimester: from 14 to 27 weeks and 6 days; and third trimester: after 28 weeks and until 40 weeks and 6 days [9]. The timing of maternal infection during gestation was based on the reporting of the first signs and symptoms suggestive of ZIKV infection, as per the manifestations described by the Brazilian Ministry of Health [10], with the typical rash from the disease appearing as one of the most striking signs.

### Variables

The analysis evaluated both maternal, perinatal, anthropometric, and clinical variables. The maternal variables were related to both socioeconomic conditions and gestational information, such as the numbers of pregnancies and prior spontaneous abortions, maternal hypertensive diseases, premature labor, and premature rupture of membranes. The perinatal variables included the date of birth, date of fetal or neonatal death, delivery type (i.e., vaginal or C-section), APGAR score (i.e., 1^st^ and 5^th^ minute), gestational age (i.e., as determined by last menstrual period, obstetric ultrasound before 12 weeks and the newborn’s physical examination, New Ballard Score [11], in this order [12]). Gestational age was analyzed in whole weeks, without specification of days, as recommended by the WHO [13]. The anthropometric variables included birth weight (BW) in grams (g) and head circumference (HC) in centimeters (cm) with the respective Z-scores to age and gender as per the InterGrowth21 neonatal growth chart [14].

The clinical variables were related to examinations and evaluations of the newborns that occurred after birth and up to 28 days of life, and included neuroimaging, ophthalmic, neurologic, and orthopedic exams. Imaging exams included transfontanellar doppler ultrasound in all newborns, cranial computed tomography scan and cranial magnetic resonance imaging (MRIs) with or without contrast in cases of suspected alterations, with reports provided by pediatric radiologists. The ophthalmic evaluations included indirect ophthalmoscopy conducted by pediatric ophthalmologists with experience in congenital infection in the neonatal population. Neurological evaluations were conducted by neuropediatricians and general clinical evaluation by pediatric infectious disease specialists. Orthopedic evaluations were performed by the pediatric orthopedics service when necessary following clinical referral.

### Outcomes

The primary fetal and neonatal outcomes considered in this study were based on the previously stated patterns reported in other studies, and the most up-to-date information about mother-to-child ZIKV infection [15]. All outcomes were defined when pregnancy ended or within the first 28 days of life, regardless of gestational age.

The primary outcomes included:

1. Abortion defined as the expulsion or extraction of a product of the conception with less than 500g and /or height less than 25 cm, or less than 22 weeks of gestation, with or without evidence of life [16].
2. Fetal death (Stillbirth) defined as the death of the gestational product before expulsion or its complete extraction from the mother’s body, regardless of pregnancy duration [16].
3. Neonatal death defined as a death occurring between 0 and 28 days of birth [17].
4. Small for gestational age (SGA) birth defined as a birth with birthweight below the 10^th^ percentile (or Z score < minus 1.28) as per the InterGrowth 21neonatal growth chart [14]. We have also considered the cutoff point of birth weight below the third percentile (or Z-score < minus 1.88), as evidence of more severe intrauterine growth restriction, according to the definition of Lee et al. (2003) [18].
5. Preterm birth defined as a birth with a gestational age of less than 37 weeks of pregnancy.
6. Symptomatic with microcephaly and/or cerebral atrophy defined as children with microcephaly and/or cerebral atrophy identified through clinical and neuro-imaging evaluations. Microcephaly was assessed in the first 24–48 hours of life and defined, respectively, as moderate or severe with a HC of < minus 2 to minus 3 or of < minus 3 standard deviations below the mean for sex and age as per the InterGrowth21 neonatal growth chart [19];
7. Symptomatic without microcephaly or cerebral atrophy defined as children with other signs and symptoms consistent with congenital ZIKV infection as described by other authors [15] and identified through the neuroimaging, ophthalmic, neurologic, and orthopedic exams;
8. Asymptomatic defined as children with normal morphological and functional evaluations within the first 28 days of life.

### Statistical analysis

The statistical analysis was performed with the program EPI-INFO version 7.2; all p-values were calculated for 2-sided statistical tests using a 0.05 level of significance. Descriptive analysis was conducted to estimate frequencies of adverse outcomes. Continuous variables were summarized using mean and standard deviations or median and interquartile range, depending on the distribution of the data and analysis of proportions to discrete or categorical variables. The prevalence of adverse outcomes was estimated by the gestational period in which the viral infection occurred. Chi-squared tests were used to compare proportions between categories; Fisher’s exact tests were used for variables with cells including 5 or fewer observations.

## Results

**Fig 1:**
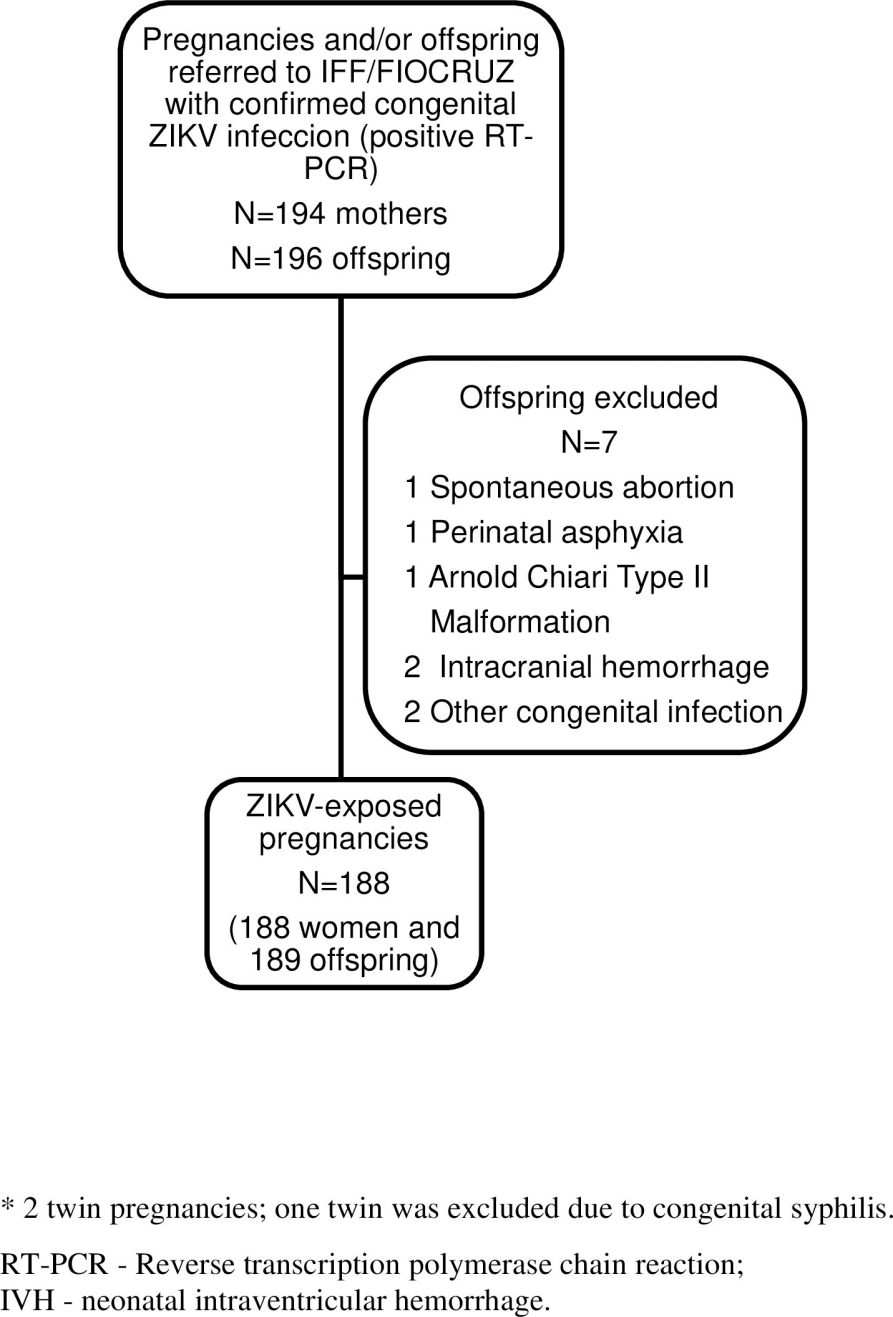
Strobe Flowchart for selection of study subjects. There are three twin pregnancies in the sample.

Of the 191 pregnancies, 194 offspring had positive RT-PCR results for ZIKV in any studied biological material (Fig 1). After one exclusion, a newborn with Arnold Chiari Syndrome, the total study population, which included three twin pregnancies, consisted of a total of 193 exposed fetuses and newborns.

Overall, the participating women had a mean age of 29.5 years (SD = 6.8), varying from 14 to 45 years. Most of these women had attained at least high school (63.1%), 55% self-reported as white, and the average monthly Family income (Brazilian Reais) was 3,400 (SD = 3,840). The primary perinatal data of the studied population are shown in Table 1. There were few co-morbidities, such as maternal hypertension (13,3%) and gestational diabetes (3,9%). Among the neonates, 69,8% were delivered by Caesarean section, and the majority (84.3%) were delivered at term with a mean gestational age of 38.2 weeks (Table 1).

**Table 1:** Demographic and perinatal characteristics of the offspring (N = 193).

VARIABLE	N	MEAN (SD)	N (%)
Multiple gestation	193		3 (1.5)
Maternal hypertension	181		24 (13.3)
Maternal diabetes	180		7 (3.9)
Maternal TORCH coinfection	193		6 (3.1)
Preterm birth	193		30 (15.5)
Male sex	191		95 (49.7)
Cesarean section	189		132 (69.8)
Gestational age at delivery (weeks)	193	38.2 (2.9)	
HC at delivery (cm)	189	33.3 (3.2)	
HC at delivery (Z-score)	189	0.17 (2.5)	
Birthweight (g)	192	2976.14 (709.3)	

TORCH—Toxoplasma gondii, others (Listeria monocytogenes, treponema pallidum, varicella-zoster virus, human immunodeficiency virus, enterovirus and parvovirus B19), rubella virus, cytomegalovirus, and herpes simplex virus. HC–head circumference.

The diferences in the sample number is due to missing values. There was one abortion from whom there weren’t information on sex and anthropometry; and one stillbirth, accounting for the lack of information on sex.

**Fig 2:**
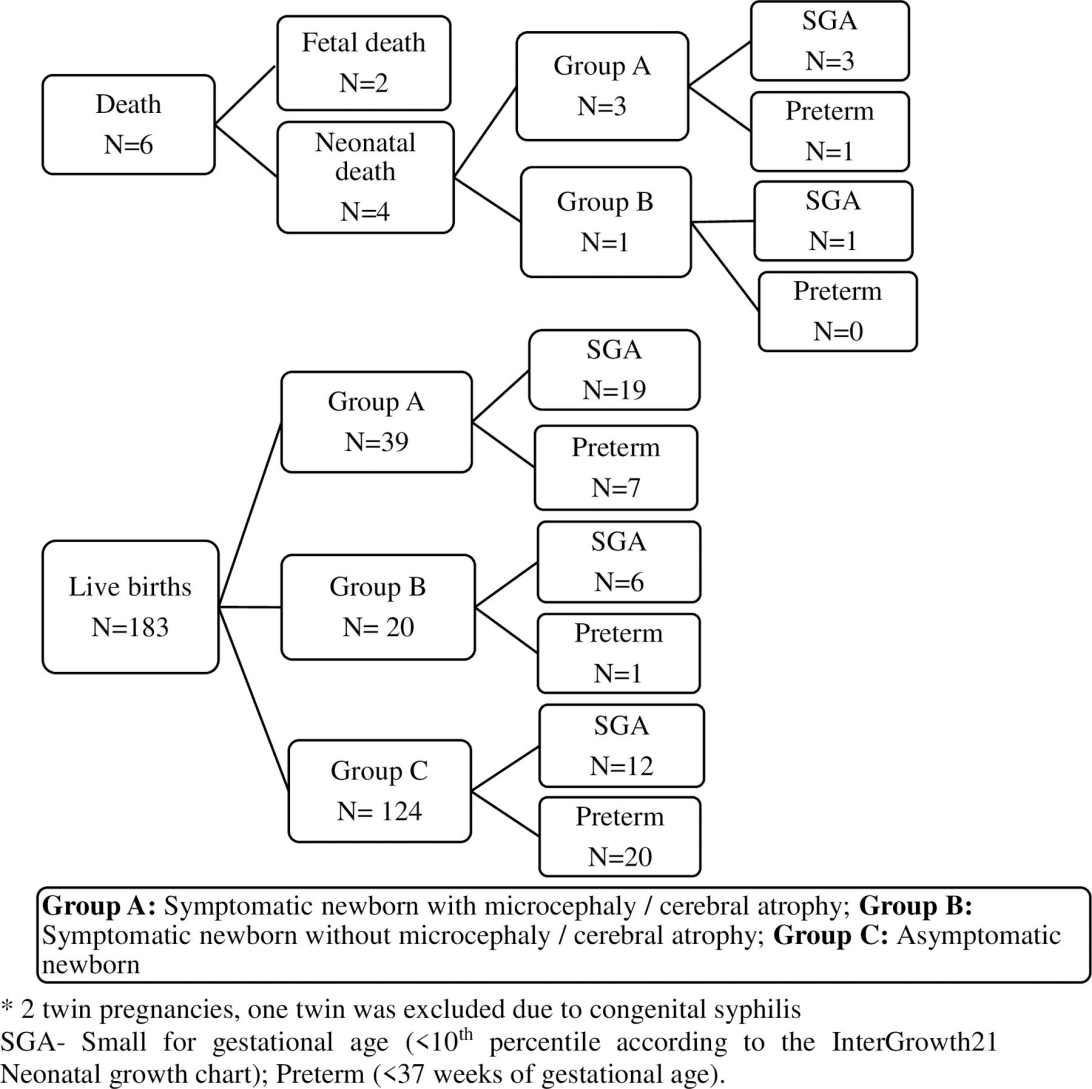
Flowchart of study outcomes (N = 191^a^). ^a^ Excluding one miscarriage and one stillbirth. SGA- Small for gestational age (<10^th^ percentile according to the InterGrowth21 Neonatal growth chart); Preterm (<37 weeks of gestational age). SGA and preterm categories are not mutually exclusive. Group A: Symptomatic with microcephaly and/or cerebral atrophy; Group B: Symptomatic without microcephaly or cerebral atrophy; Group C: Asymptomatic.

Adverse fetal and neonatal outcomes were identified in 72 offsprings (37.3%) of the 193 exposed fetus, with one abortion and one stillbirth. There were four neonatal deaths until 28 days of life; in all four cases, the mothers had a viral infection in the first gestational trimester. From the 193 exposed fetus, 70 (36.6%) presented with abnormalities upon examination and 121 offspring, representing 63.3%, were asymptomatic upon evaluation. Among the 70 symptomatic neonates at birth, 41 had microcephaly and/or cerebral atrophy (58.6%), and 29 neonates (41.4%) had other characteristics suggestive of ZIKV congenital disease, as described by Moore et al [15], without microcephaly or cerebral atrophy. (Fig 2). Among the neonatal deaths, three were born with evidence of microcephaly and/or cerebral atrophy and one only presented with the characteristics of congenital ZIKV infection but without microcephaly. All four neonatal deaths were considered small for gestational age, and one was delivered prematurely. Microcephaly in the full study population totaled 36 cases (18.7%), of which 27 cases (14.1%) were classified as severe microcephaly.

Out of the 184 pregnancies with known period of maternal ZIKV symptoms, 64 (34.8%), 85 (46.2%), and 35 (19.0%) women reported the symptoms in the first, second, and third trimesters of pregnancy, respectively. In nine cases, the mothers could not report the period of infection. The symptomatic newborns, with and without microcephaly, were exposed predominantly in the first trimester, while those born asymptomatic were exposed mainly in the second trimester (Table 2). Also, the SGA newborns were predominantly exposed in the first trimester. In this population, 29 newborns were preterm (15.0%), of which 26 (89,6%) were classified as moderate preterm (i.e., between 32 weeks and 36 weeks and 6 days of gestational age).There was no significant difference in the trimester of gestation exposition for premature birth (Table 2).

**Table 2:** Relationship between the trimester of infection and the clinical outcomes (N = 182^b^).

		GESTATIONAL TRIMESTER N (%)			
OUTCOME	N	1st	2nd	3rd		p-value^a^
**GROUP A**	35	29 (82.9)	6 (17.1)	0		<0.0001
**GROUP B**	28	13 (46.4)	10 (35.7)	5 (17.9)		<0.0001
**GROUP C**	119	21 (17.6)	68 (57.1)	30 (25.2)		<0.0001
**SGA**	38	21 (33.3)	15 (17.6)	2 (5.7)		0.003
**Premature**	28	10 (15.9)	13 (15.3)	5 (14.3)		0.978

**a** Pearson Chi Square was used to compare the distribution of the groups, the prevalence of SGA and the prevalence of premature in relation of the trimester of gestation.

SGA- Small for gestational age (<10^th^ percentile on InterGrowth21 Neonatal growth chart).

Group A: Symptomatic with microcephaly and/or cerebral atrophy; Group B: Symptomatic without microcephaly or cerebral atrophy; Group C: Asymptomatic.

**b** Exclusion from the analysis: nine cases with uncertain trimester of exposition, one abortion and one stillbirth in which the presence of microcephaly was not possible to confirm.

In the study population, 42 of 193 deliveries (21.9%) were SGA (i.e., less than the tenth percentile). There were 12 offspring who were both SGA and premature at delivery (i.e., 6.2% of deliveries overall). The distribution of SGA cases also varied by the phenotype of liveborn neonates, with of 31/42 SGA cases (73.8%) exhibiting symptoms consistent with congenital ZIKV infection (Fig 2). Considering a more restrictive cutoff point to define SGA, 38 (90.4%) of the 42 SGA deliveries fell below the third percentile. In this sample, 58.5% of the cases classified in Group A (i.e., symptomatic with microcephaly/cerebral atrophy) and 24.1% classified in Group B (i.e., symptomatic without microcephaly/cerebral atrophy) were SGA (Table 3). Notably, there were 23 SGA under the third percentile in Group A.

**Table 3:** Association of the offspring congenital Zika virus infection symptomatology with frequency of SGA, also considering a more restrictive cutoff point (N = 191).

	GROUP A n (%)	GROUP B n (%)	GROUP C p-value n (%)	
SGA < p10	24 (58.5)	7 (24.1)	11 (9.1)	<0.0001
SGA < p3	23 (59.0)	5 (17.9)	10 (8.3)	<0.0001

SGA-Small for gestational age, below the 10^th^ (P10) and 3^rd^ percentile (P3), respectively, on InterGrowth21 Neonatal growth chart.

Group A: Symptomatic with microcephaly and/or cerebral atrophy; Group B: Symptomatic without microcephaly or cerebral atrophy; Group C: Asymptomatic.

The clinical presentation of neonates with symptoms consistent with congenital ZIKV infection were significantly associated with the first trimester of exposition to ZIKV. The most severe abnormalities were predominantly related to the first trimester infection, although the exposition in the second trimester was also associated with the impairment of fetus development (Table 4). Microphtalmia and Coloboma were not related to the trimester of infection, though the numbers of affected children were very small (Table 4).

**Table 4:** Common anomalies associated with congenital Zika Virus infection in this sample of symptomatic offspring per trimester of gestation (N = 184).

CHARACTERISTICS	N	1 st TRIMESTER n (%)	2 nd TRIMESTER n (%)	3rd TRIMESTER n (%)	p-value
**CRANIAL MORPHOLOGY**					
Microcephaly	182	24 (38.1)	6 (7.1)	0	<0.0001
**BRAIN ANOMALIES**					
Cerebral cortex thinning	171	27 (46.6)	6 (7.3)	0	<0.0001
Abnormal gyral patterns	171	26 (44.8)	6 (7.3)	0	<0.0001
Corpus callosum anomalies	171	8 (13.8)	5 (6.1)	0	0.05
Cerebellar hypoplasia	171	8 (13.8)	1 (1.2)	0	0.002
Decreased white matter	171	11 (20.4)	4 (4.9)	0	0.001
Ventriculomegaly	170	29 (37.9)	3 (3.7)	0	<0.0001
Subcortical calcification	171	32 (55.2)	5 (6.1)	0	<0.0001
**MUSKULOSKELETAL ANOMALIES**					
Congenital clubfoot	179	3 (4.9)	0	0	0.052
Arthrogryposis	179	6 (9.8)	2 (2.4)	0	0.038
Hip dislocation	179	5 (8.2)	1 (1.2)	0	0.033
**NEUROLOGICAL IMPAIRMENT**					
Motor disability	175	31 (53.4)	10 (12.0)	1 (2.9)	<0.0001
Hypertonia	175	32 (55.2)	9 (10.8)	3 (8.8)	<0.0001
Pyramidal syndrome	175	29 (50.0)	7 (8.4)	2 (5.9)	<0.0001
Hyperexcitability	175	21 (36.2)	6 (7.2)	0	<0.0001
Epilepsy	175	27 (46.6)	6 (7.2)	0	<0.0001
Swallowing dysfunction	175	13 (22.4)	1 (1.2)	1 (2.9)	<0.0001
**OPHTALMOLOGICAL ANOMALIES**					
Microphthalmia	173	1 (1.8)	0	1 (2.9)	0.352
Coloboma	173	1 (1.8)	2 (2.4)	1 (2.9)	0.931
Chorioretinal atrophy	173	16 (28.1)	4 (4.9)	0	<0.0001
Pigment mottling	173	12 (21.1)	1 (1.2)	0	<0.0001
Optic nerve hypoplasia	173	15 (26.3)	2 (2.4)	0	0.0001

Pearson Chi Square test.

11 offsprings were excluded: 9 with uncertain period of infection during gestation and 2 fetal deaths (one miscarriage and one stillbirth due to the impossibility of clinical investigation.

In 14 newborns there were muskuloskeleton anomalies. In 49 there were neurological abnormalities and 173 had an ophtalmological evaluation.

List of features modified from Moore et al–Characterizing the pattern of anomalies in Congenital Zika Syndrome for pediatric clinicians. JAMA Pediatric.2017;171(3):288–295 (15).

## Discussion

This study recruited the pregnant women during the outbreak of ZIKV infection which occurred in Brazil between 2015–2017. This study showed that the earlier the symptoms appear during gestation, the more severe the endpoints. About half of the patients complained of viral infection signs and symptoms during the second trimester, which is in line with what it was reported by Van Maldeghem K et al. [5] and França V A et al [20,1]. Within adverse pregnancy endpoints, neonates that were symptomatic with microcephaly/cerebral atrophy, small for gestational age (SGA) and stillborn were more common in women that had symptoms earlier during their pregnancy, which confirms data previously described (3–5) [21]. Microcephaly was present in 21.2% of the offsprings, which is significantly higher when compared to the systematic review of Campos Coelho A. V. et al. (2017) [21], which reported a range from 1 to 13%, depending on location, to the 1% for French Polynesia [22] and to the 13% for the state of Bahia [23]. However, our institution is a reference for malformations and received pregnant women or newborn after birth with malformations and this can explain high rate of microcephaly. Previous results from the same cohort in Rio de Janeiro, including only the pregnant women with symptoms (fever and cutaneous rash) and positive RT-PCR results, published recently, described only four cases of microcephaly (3.4%) (2). Once again, most microcephaly cases were related to maternal viral infection during the first trimester, as occurred in our sample. Nonetheless, our high incidence of microcephaly could be explained because the hospital is a referral center for congenital abnormalities during pregnancy. Our study showed no case of microcephaly associated with maternal viral infection during the third trimester.

Our research found that 37.3% of pregnancies with exposition to ZIKV resulted in fetal or neonatal adverse outcomes. In the literature, these proportions range from 5% [5] to 6% [4] in two North American studies, 7% in French American territory (3), 55% in the French Guiana [6]. A case control study in the Northeast of Brazil confirmed the association of microcephaly with ZIKV exposure during gestation and refers 43% of association of cerebral abnormalities with ZIKV infection in positive cases [24] The studies cited employed different methodologies, related to the availability of imaging tests, selection of the exposed, and evaluation of the newborns, which can explain the variation of the percentages found, much lower than our study. One of the explanations for this finding could be related to the more detailed investigation of the newborns in our study, with more sophisticated imaging tests performed in every altered case, with cranial MRIs and CT scans, as well as multi-professional evaluation until 28 days of age. Another explanation is that this was a reference hospital for ZIKV infection in pregnant women and newborns, and also in fetal malformations in general.

The proportion of prematurity in this study was similar to the world percentage, about 11% according to WHO [12], and 11.5% in Brazil according to Birth in Brazil research [25], as well as in the population exposed to ZIKV: 13% in French Guiana [6] and 8.8% in New York City [26]. Therefore, it could be assumed that mother-to-child ZIKV infection does not lead to higher levels of premature births.

We found a higher percentage of small for gestational age newborns (21.9%) compared to the Brazilian population and to other studies concerning the newborn population exposed to ZIKV during gestation [3,26,27,28,29]. The study by Lee et al. (2013), including the population of several underdeveloped and developing countries, reported a prevalence of 12.5% in Latin America and the Caribbean [27], while in the population exposed to ZIKV, the proportions ranged from 9% [2] to 13% [3], using the same definitions proposed by this study. The comparison of the prevalence of SGA newborns in Brazil is limited since national studies include different profiles of hospital complexity, specific populations from each region and the use of diverse methods for the classification of SGA newborns. In our study, the proportion of SGA newborns was as significant as the more severe involvement of newborns, which is in agreement with the study by Prata-Barbosa et al. [28]. The same trend occurred concerning the distribution throughout gestational trimesters: the earlier the maternal infection, the higher the percentage of SGA newborns. Others studies with ZIKV-infected mothers, considering the same definition for IUGR, reported a prevalence of SGA of 11.2% in Cooper et al [26], 12% in French Guiana [6] and 18% in Walker CL et al. [29], who also found head and abdominal circumferences below the 10^th^ percentile in the ultrasounds performed. Thus, it could be inferred that symmetrical IUGR in fetal ultrasounds might be an early gestational marker of a newborn probably vertically infected by ZIKV.

The referred population to this facility during the Zika epidemic originated from both private and public services, which could justify the higher maternal income and education level of the studied population.

The strengths and limitations of the study warrant consideration. The key strength of the study is that affected newborns were evaluated through specialized clinical investigations and accurate imaging tests of the central nervous system. Cranial CT scans and MRIs were made in almost every affected neonate, which also contributed to the identification of a more significant number of the alterations found. One limitation of this study was that the inclusion of some women after the fetal ultrasound with mycrocephaly. These fetal outcomes were detected during the routine ultrasound in asymptomatic women and they were included in the recruitment process, when previous fetal development impairment could not be known for certain, so possibly introducing a selection bias. However, this was a reference hospital for ZIKV infections in pregnant women during an epidemic, and they were referred because, although not having symptoms of ZIKV infection, they were tested after a fetal ultrasound showing abnormalities, and they had a positive RT-PCR in any biological material. Another limitation was the size of the sample, which did not allow us to evaluate the occurrence of SGA adjusting for others causes, for example, maternal hypertension, as we had only 13.3% of them in this sample. We are also not describing a control group in this paper.

## Conclusions

The study confirmed the already reported relationships in other papers, which is the earlier the ZIKV infection during pregnancy, the more severe the fetal endpoints and, therefore, the greater morbimortality in the affected subjects. The higher percentage of SGA newborns found in this study deserves attention, not only for the adequate management of the affected population during the epidemics, but also the investigation of its causal mechanisms. This study also identified a group of neonates without microcephaly but who presented with other anomalies, neurological or clinical, and furthermore, we also identified another group that although having a proven exposition during pregnancy were born apparently normal. This is a matter of concern, as one cannot predict if these babies will remain with a normal development in future. A long-term longitudinal study must be considered to evaluate better the endpoints brought by this congenital infection, especially the asymptomatic newborns, which could help the understanding of the long-term consequences of this infection in pregnant women.
